# Importance of Michaelis Constants for Cancer Cell Redox Balance and Lactate Secretion—Revisiting the Warburg Effect

**DOI:** 10.3390/cancers16132290

**Published:** 2024-06-21

**Authors:** Michael Niepmann

**Affiliations:** Institute of Biochemistry, Medical Faculty, Justus-Liebig-University, 35392 Giessen, Germany; michael.niepmann@biochemie.med.uni-giessen.de

**Keywords:** Warburg effect, anaerobic, aerobic glycolysis, glucose, pyruvate, lactate, lactic acid, cancer, tumor, K_M_ value, Michaelis constant

## Abstract

**Simple Summary:**

In cancer cells, gross changes in gene expression help to enhance the uptake of glucose—the blood sugar—for the increased formation of building blocks for the cancer cells’ unlimited growth. An issue related to the rewired cancer cell metabolism is that even under sufficient oxygen supply, cancer cells metabolize a large fraction of the glucose to lactic acid (or “lactate”). In non-cancerous cells, lactate would be formed only during oxygen deprivation in order to re-oxidize the cytosolic electron carrier NADH, which forms during the glycolytic glucose breakdown. This phenomenon of lactate secretion from cancer cells under aerobic conditions was named the “Warburg Effect”, but the actual reasons for that are often regarded as not yet understood. However, when we acknowledge that the reprogramming of the different metabolic pathways of cancer cells is not neatly fine-tuned, it becomes plausible that lactate formation just serves to dispose of cytosolic electrons that exceed the capacity of the mitochondrial electron transport chain to accept cytosolic electrons. Interestingly, the kinetic properties of the enzymes that metabolize the glycolysis end product pyruvate are sufficient to explain the priorities for metabolite flux at the pyruvate junction in cancer cells: 1. mitochondrial oxidative phosphorylation for efficient ATP production, 2. cytosolic electrons that exceed oxidative phosphorylation capacity need to be disposed of and secreted as lactate, and 3. biosynthesis reactions for cancer cell growth. In other words, a number of cytosolic electrons just take the “emergency exit” from the cell by lactate secretion to maintain the cytosolic redox balance.

**Abstract:**

Cancer cells metabolize a large fraction of glucose to lactate, even under a sufficient oxygen supply. This phenomenon—the “Warburg Effect”—is often regarded as not yet understood. Cancer cells change gene expression to increase the uptake and utilization of glucose for biosynthesis pathways and glycolysis, but they do not adequately up-regulate the tricarboxylic acid (TCA) cycle and oxidative phosphorylation (OXPHOS). Thereby, an increased glycolytic flux causes an increased production of cytosolic NADH. However, since the corresponding gene expression changes are not neatly fine-tuned in the cancer cells, cytosolic NAD^+^ must often be regenerated by loading excess electrons onto pyruvate and secreting the resulting lactate, even under sufficient oxygen supply. Interestingly, the Michaelis constants (K_M_ values) of the enzymes at the pyruvate junction are sufficient to explain the priorities for pyruvate utilization in cancer cells: 1. mitochondrial OXPHOS for efficient ATP production, 2. electrons that exceed OXPHOS capacity need to be disposed of and secreted as lactate, and 3. biosynthesis reactions for cancer cell growth. In other words, a number of cytosolic electrons need to take the “emergency exit” from the cell by lactate secretion to maintain the cytosolic redox balance.

## 1. Introduction

In the 1920s, Otto Warburg, as well as Gerty and Carl Cori, conducted experiments that revealed the metabolization of glucose to lactic acid (also called lactate, the anion of lactic acid) in tumor cells even under sufficient oxygen supply [1,2,3,4,5,6]. Later, this effect of “aerobic glycolysis” was called the “Warburg effect” [7,8]. This observation contrasted Warburg’s findings that normal (non-tumor) tissue produced lactate essentially only under conditions of oxygen deprivation. Warburg originally claimed this effect (which he attributed to impaired respiration) to be a cause of cancer [6,8]. However, nowadays, we know that essentially genetic and gene expression changes that facilitate uncontrolled cell growth cause cancer [9,10,11,12], while the production of considerable amounts of lactate, even in the presence of sufficient amounts of O_2_—“aerobic” or “normoxic” glycolysis—is supposed to be a consequence of such gene expression changes rather than their cause [9,11,13,14].

Even though for many tumor cells (mostly in solid tumors) short supply of oxygen can also be an important issue due to insufficient vascularization and limitations in O_2_ diffusion [15,16,17], tumor cells routinely also display the Warburg effect under conditions of sufficient oxygen supply. Notably, the tricarboxylic acid (TCA) cycle (also named citric acid cycle or Krebs cycle), as well as the electron transport chain (ETC) and oxidative phosphorylation (OXPHOS), have been shown to be active in many cancer cells [13,14,18,19,20,21,22], as it is also the case in other highly proliferating cells, such as lymphocytes [23]. Actually, the cells even require ongoing oxidative phosphorylation for efficient proliferation [19,24], since they can produce much more ATP in the presence of sufficient O_2_ [25].

In this review, I will focus on the need for tumor cells to secrete lactate even in the presence of oxygen, i.e., the actual “Warburg effect”. Even though a few particular gene expression changes are actually involved in causing the metabolic effects of the Warburg effect itself, I will only briefly touch on those gene expression changes that actually cause cancer, but rather focus on the mechanistic aspects of the key enzymes at the pyruvate junction—the point where several metabolic pathways branch from the principal first end product of glycolysis, pyruvate. In combination with some very limited changes in the expression of some of these key enzymes, mainly their enzymatic properties tell us what the priorities for the use of glucose in cancer cells are, and thereby fully explain the supposed enigma of why cancer cells secrete lactate. It is all about maintaining the cytosolic redox balance in an environment in which rather coarse gene expression changes have rewired the carbon metabolite flux in the cancer cells in order to support their unlimited growth.

As a basis for understanding, I will first give a brief overview of glucose metabolization and lactate production, as well as the consumption of lactate in normal (non-cancer) body tissues (Section 2), with a focus on the enzymes at the pyruvate junction that play a role in the production and utilization of the key metabolite pyruvate (Section 3). Then, I will consider the gene expression and metabolic changes in tumor cells in Section 4, which will be just a brief overview, since many excellent studies and reviews have explained these aspects in detail. Finally, I will return to the key enzymes at the pyruvate junction and explain their properties, largely in terms of their Michaelis constants (K_M_ values), an aspect that may have been underestimated in the literature about the Warburg effect. Just by careful inspection of their kinetic properties (in addition to the aspects of altered gene regulation), these enzymes tell us what the most important metabolic pathways deriving from the pyruvate junction are (Section 5). Based on the results, it becomes clear that the regeneration of ATP by the TCA cycle and OXPHOS is the first priority, while the disposal of overflow electrons via the generation of lactate just serves to maintain the cytosolic redox balance under the conditions of the coarse gene expression deregulation in cancer cells.

## 2. Glucose and Lactate Metabolism in Normal Body Tissues

**Figure 1:**
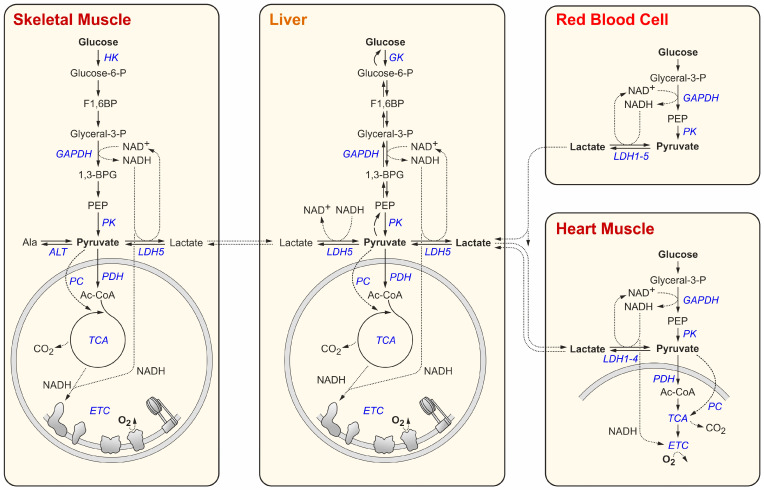
Glucose utilization in non-cancerous cells (skeletal and heart muscle, liver, erythrocyte). This view is simplified to allow readers to focus on the metabolization of glucose and the production, as well as the consumption, of lactate in the tissues. Abbreviations: HK, hexokinase; GAPDH, glyceraldehyde-3-phosphate dehydrogenase; PK, pyruvate kinase; PDH, pyruvate dehydrogenase; PC, pyruvate carboxylase; ALT, alanine transaminase; LDH, lactate dehydrogenase; TCA, Tricarboxylic acid cycle (or Krebs cycle); ETC, electron transport chain (i.e., respiratory chain). Glucose-6-P, glucose-6-phosphate; F1,6BP, fructose-1,6-bisphosphate; Glyceral-3-P, glyceral(dehyde)-3-phosphate; 1,3-BPG, 1,3-bisphosphoglycerate; PEP, phosphoenolpyruvate; Ala, alanine; Ac-CoA, acetyl-coenzyme A; NAD^+^, nicotinamide adenine dinucleotide; NADH, reduced form of NAD^+^.

Glucose is a standard C_6_ carbon source for virtually all tissues in the body and is broken down by glycolysis [26]. On the way from glucose to pyruvate, glucokinase (GK) in liver and pancreatic β-cells and hexokinase (HK) in virtually all other cells phosphorylate glucose to glucose-6-phosphate (Figure 1). After two further conversion steps, the C_6_ body fructose-1,6-bisphosphate (F1,6BP) is formed, which then is cleaved into two C_3_ bodies, which yield two molecules of glyceraldehyde-3-phosphate (or glyceral-3-phosphate). The next step is catalyzed by glyceraldehyde-3-phosphate dehydrogenase (GAPDH). This step is important, since it oxidizes the substrate and transfers a hydride ion (H^−^, i.e., one proton coming with two electrons) from the substrate to the electron carrier NAD^+^, reducing it to NADH. The C_3_ bodies are further converted to phosphoenolpyruvate (PEP). The last step then converts PEP to pyruvate and is catalyzed by pyruvate kinase (PK). In the entire flow of glycolysis, per one C_6_ molecule of glucose, two ATP molecules are generated by substrate-level phosphorylation, i.e., without the requirement for O_2_.

In most tissues, the end product of glycolysis, pyruvate (C_3_), can be used by the mitochondrial pyruvate dehydrogenase (PDH) to generate acetyl-Coenzyme A (Ac-CoA), which delivers its C_2_ acetyl residue to the TCA cycle, where both C atoms are finally released in their maximally oxidized form as two molecules of CO_2_ [27]. The electrons yielded by the stepwise oxidation of the moderately reduced C atoms from glucose are transported by electron carriers such as NADH to enter the mitochondrial ETC and are finally transferred to O_2_ as the final electron acceptor (Figure 1). In summary, they yield about 32 ATP per one C_6_ glucose by all steps, including oxidative phosphorylation.

**Figure 2:**
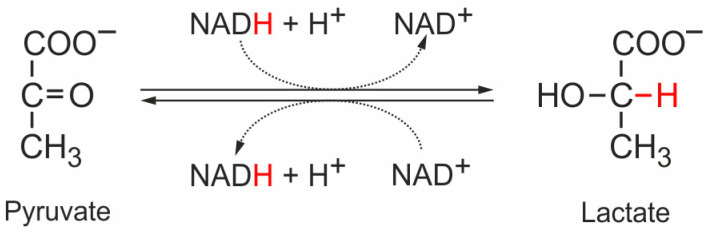
The lactate dehydrogenase reaction. NADH provides a hydride ion that is transferred to the C atom of the carbonyl group of pyruvate. Thereby, the two electrons of the hydride ion provide both electrons of the H-C bond in the resulting lactate (in red). The C=O double bond of pyruvate switches one electron pair outside, which then acquires a proton from the solution, forming the hydroxyl group in lactate. In short, NADH is a two-electron carrier and transfers these two electrons to pyruvate, forming lactate [26,30,31].

Only in erythrocytes (red blood cells, RBCs), there are no mitochondria, for the obvious reason that the RBCs are supposed to transport the O_2_ from the lungs to peripheral tissues instead of using up the O_2_ for their own energy demands. In this case, the two electrons from the NADH generated in the GAPDH reaction need to be disposed of in some other way. Otherwise, NAD^+^ levels would decrease quickly, and glycolysis would stop. Therefore, in RBCs this electron disposal problem is solved by transferring the two electrons from NADH to the end product of glycolysis, pyruvate, to form lactate in the lactate dehydrogenase (LDH) reaction (Figure 2). The lactate must then be secreted from the erythrocytes (Figure 1). Similarly, muscle cells that sometimes need to work intensely, even under insufficient oxygen supply conditions, can use this “emergency exit” for the electrons from cytosolic NADH and form lactate from pyruvate in order to keep glycolysis running (Figure 1). The lactate secreted from the muscle—similar to the lactate from erythrocytes—can then be used by the liver (or by kidney) [28] to first form pyruvate and then glucose in the process of gluconeogenesis, and the liver can then provide this glucose again for the peripheral tissues. This glucose–lactate cycle is called the Cori cycle [29].

Lactate can be consumed not only by the liver but also by the heart muscle, which is the muscle with the likely best supply of oxygen. Therefore, the heart muscle can oxidize lactate and use the resulting pyruvate as a carbon source for the TCA cycle. For this reason, the heart muscle cells express LDH-1 or closely related isoforms [32,33,34], of which some have low K_M_ values and therefore ensure efficient use of lactate from the blood, depending on the blood lactate concentrations, which can largely vary according to the body´s energy consumption conditions (rest vs. exercise) (see Table 1 and references therein). Since LDH catalyzes a reversible reaction and, similar to virtually all enzymes, only accelerates the reaction but does not shift the equilibrium, it is not surprising that extracellular and intracellular lactate and pyruvate concentrations appear in quite similar ratios (Table 1).

Remarkably, the concentrations of pyruvate and lactate were reported to be higher in the cancer cell lines than in the red blood cells (Table 1). However, in these studies the metabolite concentrations were analyzed only in cancer cells but not in normal tissues for comparison. The increased lactate and pyruvate concentrations may support cancer cell growth by also increasing the concentrations of upstream glycolytic metabolites. Since the LDH supports the maintenance of the intracellular lactate/pyruvate ratio, the increase in intracellular lactate concentration may also be due to changes in the expression of lactate transporters in the cancer cells. Accordingly, the monocarboxylate transporters MCT-1 and MCT-4, which have higher K_M_ values for lactate compared to MCT-2 and MCT-3, are upregulated in many cancer cells [35,36,37,38,39,40,41], thereby supporting the maintenance of higher concentrations of lactate and upstream metabolites in the cancer cells.

**Table 1:** Key metabolite concentrations in blood, and intracellularly in red blood cells and tumor cell lines.

Sample	Metabolite	Concentration (mM)				Ratio	References
		Means	Range ^1^	SD	*n* ^2^		
Blood	lactate (resting)	1.597	0.800–3.500	0.738	10	[lac]/[pyr] 22.0	[42,43,44,45,46,47,48,49,50]
	pyruvate (resting)	0.073	0.032–0.120	0.027	7		[46,47,48,49,50,51,52]
	lactate (exercising)	6.200	5.500–7.500	0.748	5	-	[45,47,49,50,52]
RBC	lactate	1.021	0.200–1.870	0.454	7	[lac]/[pyr] 16.7	[43,44,46,53,54,55]
	pyruvate	0.061	0.043–0.083	0.013	5		[46,53,54,55]
	NAD^+^	0.051	0.040–0.062	0.011	2	[NAD^+^]/[NADH] 1.9	[46,55]
	NADH	0.027	0.027	0.000	1		[55]
Tumor cell line	lactate	13.033	2.000–35.525	10.905	12	[lac]/[pyr] 17.8	[39,56,57]
	pyruvate	0.733	0.130–5.880	1.326	19		[56,57,58,59,60]
	NAD^+^	0.486	0.470–0.502	0.016	2	[NAD^+^]/[NADH] 6.9	[58,59]
	NADH	0.070	0.065–0.075	0.005	2		[58,59]

^1^ As found over all the various references. Detailed values and additional information are shown in Supplementary Table S1. ^2^ Number of independent experiments from references.

Nevertheless, all tissues (except for RBCs) that express LDH can either consume or produce lactate [61]. The latter is—in contrast to widespread assumptions—also true for the heart muscle [62,63] which can also secrete lactate, depending on both internal and external lactate and pyruvate concentrations and (limiting) oxygen supply. Recent tracer studies have shown that the carbon atoms provided by fed glucose appear in lactate (also produced by the heart muscle) and in other downstream metabolites. In turn, lactate is also routinely utilized by virtually all tissues to feed the TCA cycle and other pathways [28,64,65,66,67,68,69,70] (see Figure 1). Moreover, lactate can even stimulate its own use by mitochondria by yet unknown mechanisms, independent of its actual use [69]. In short, lactate can be produced by virtually all tissues, and it can be consumed by all tissues that have mitochondria.

## 3. Key Enzymes and Metabolites at the Pyruvate Junction

For understanding the utilization of carbon backbones downstream of glycolysis in normal (non-cancerous) tissues, it appears useful to have a look at the properties of the enzymes at the pyruvate junction, i.e., the enzymes that produce pyruvate and then convert it to other metabolites. The Michaelis constants (or K_M_ values) of enzymes are a reciprocal measure for the affinity of the enzyme to the substrate. The K_M_ value is the substrate concentration at which the velocity (v_0_) of the reaction is ½ of the maximal velocity (v_max_), and a low K_M_ indicates a high substrate affinity, whereas a high K_M_ indicates a low substrate affinity.

We can first consider pyruvate kinase, which converts PEP to pyruvate. PK comes in different isoforms [71], and some PK isoforms (including PKM2) undergo complex regulation by allosteric effectors, as well as phosphorylation of the enzyme [71,72,73,74]. PKM1 expressed in the muscle and the brain is not stimulated by the upstream glycolytic metabolite F1,6BP but by default has a high affinity for its substrate PEP, with a low K_M_ of about 0.057 mM (see Table 2). Thus, under energy demand conditions PKM1 keeps glycolysis running to feed the PDH and the TCA cycle reactions. In contrast, in other tissues, including the liver, the erythrocytes, and many others, either the PKL/R or the PKM2 isoforms are expressed. These have moderately high K_M_ values of 0.42 to 0.74 mM for PEP in the absence of F1,6BP (Table 2), while 1 mM of the allosteric activator F1,6BP strongly reduces the K_M_ values to about 0.07–0.08 mM [75], i.e., increases the affinity of the enzymes for PEP by about 10-fold (Table 2). This regulation by F1,6BP may be required to allow a certain extent of accumulation of F1,6BP and the upstream glucose-6-phosphate, since glucose-6-phosphate can be used also for the pentose phosphate pathway (PPP), which is required particularly in RBCs for the NADPH-dependent production of glutathione and in hepatocytes for various biosynthesis reactions involving riboses and/or NADPH.

When we consider the reactions that utilize pyruvate, pyruvate dehydrogenase has a very low K_M_ of about 0.02 mM for pyruvate (Table 2). This is by far the lowest K_M_ of all enzymes utilizing pyruvate, and it means that the PDH reaction is, in principle, overriding all other pyruvate consuming reactions. The PDH activity can be inhibited by PDH kinases (PDKs) and stimulated by PDH phosphatases (PDPs), with PDKs activated by acetyl-CoA, NADH, and ATP (i.e., then finally inhibiting PDH activity), and PDKs can be inhibited by pyruvate, CoA, NAD^+^, and ADP (i.e., then finally allowing activation of PDH) [126]. In short, PDH is activated by substrate availability and energy demand but inhibited by its own products, by ATP and by NADH. Thus, due to its extremely low K_M_ for pyruvate, PDH must be regarded as the principal gate keeper for the use of pyruvate towards the TCA cycle and oxidative phosphorylation.

All other enzymes at the pyruvate junction have much lower affinities for pyruvate compared to PDH (Table 2). Pyruvate carboxylase (PC), which catalyzes the anaplerotic production of oxaloacetate to refill the TCA cycle (Figure 1), has a K_M_ of 0.265 mM for pyruvate. This means that pyruvate is only used to refill oxaloacetate into the TCA cycle when pyruvate accumulates because the TCA cycle is slowed down due to the deprivation of its metabolites. Moreover, alanine aminotransferase (or alanine transaminase, ALT) has an even much higher K_M_ for pyruvate (about 2.8 mM). When we consider that the pyruvate concentration in the cell on average is about 0.06 mM in RBCs and about 0.73 mM in cancer cells (Table 1), this means that pyruvate is only used for the production of alanine when all other needs for the use of pyruvate have been covered.

The K_M_ of lactate dehydrogenase ranges in between those of PDH and ALT, depending on the organ (Table 2). Thereby, a tissue may contain a spectrum of different isoforms of the respective enzyme [33,34], while the enzymatic properties of each isoform are defined by a given isoform´s subunit composition, as demonstrated by using recombinant enzymes [117,118]. In the heart muscle, which, on the one hand, can efficiently use lactate as a carbon source but, on the other hand, should also be able to efficiently dispose of electrons via lactate secretion when O_2_ becomes limiting, a spectrum of LDH isoforms is expressed [33,34] (Figure 1 and Table 2), of which LDH-1 (i.e., LDH-B) has a K_M_ of only 0.1 mM for pyruvate and therefore can dispose of electrons efficiently. Erythrocytes, which must dispose of electrons via lactate routinely since they have no mitochondria, also contain a spectrum of LDH-1-5 isoenzymes [33,34], providing a range of K_M_ values. In contrast, the skeletal muscle and the liver, which should either secrete or consume lactate only above a certain threshold, essentially express LDH-5 (LDH-A), which has a higher K_M_ of about 0.29 mM for pyruvate (Table 2). The reason for that may be that the liver should routinely use the lactate from erythrocytes but keep blood lactate levels high enough for lactate to be consumed as a carbon source by the heart muscle and other organs. The skeletal muscle must be able to dispose of electrons under limiting O_2_ conditions, but it should keep an intracellular pyruvate concentration high enough to allow the use of pyruvate for many other purposes, including the TCA cycle.

## 4. Switching to the Tumor Metabolic Phenotype

In cancer cells, the underlying regulatory mechanisms that result in the metabolic reprogramming of the cell include (among others) the up-regulation of the mutated tumor suppressor p53, Myc (Myelocytomatosis oncogene), HIF1 (hypoxia-induced factor 1), NRF2 (nuclear factor erythroid 2-related factor 2) and SQSTM1 (Sequestosome 1) [39,127,128,129,130,131,132,133]. These factors then up-regulate the expression of several enzymes involved in the uptake and breakdown of glucose, including hexokinase 2 (HK2), pyruvate kinases, often including the isoform PKM2, LDH-5 (LDH-A), pyruvate dehydrogenase kinase 1 (PDK1), which can inhibit PDH activity according to metabolic requirements [39,41,126,127,129,130,131,134], as well as the MCT-1 and MCT-4 transporters mentioned above [35,36,37,38,39,40,41].

In addition to glycolysis [9,11,135,136,137], the expression of enzymes of several biosynthesis-related pathways is upregulated in cancer cells, including the PPP [9,11,22,135,136,138,139], which provides riboses for the DNA and RNA synthesis, as well as NADPH, which is required in large amounts in particular for lipid production, fatty acid synthesis [11,135,138], cholesterol synthesis [138], and amino acid synthesis [138]. Oncogenic viruses also cause the reprogramming of the cells towards a cancer cell-like phenotype [138,140].

In contrast, the situation with the expression of enzymes involved in catabolic downstream pathways for the complete carbon-body breakdown and oxidative phosphorylation, which finally provide energy, was found to be more diverse in cancer cells, perhaps also due to the amphibolic nature of the TCA cycle. Some studies showed the up-regulation of the TCA cycle and oxidative phosphorylation enzymes [9,11,19,135], whereas other studies found these pathways down-regulated or not significantly regulated [136,137,138,141,142,143]. Not down-regulating or even up-regulating TCA and OXPHOS may make sense since fast-growing cancer cells need a lot of energy, as illustrated by the finding that proliferating cancer cells can up-regulate these pathways [19].

As described above, lactate dehydrogenase isoenzymes differ by their expression among normal body tissues and by their affinities to their substrates. The LDH-1 expressed in the heart and in red blood cells (RBCs) has a moderately low K_M_ value of about 0.1 mM for pyruvate. This allows the efficient disposal of lactate from RBCs, which obligatorily secrete lactate because they do not have mitochondria. LDH-1 also allows the efficient removal of lactate (i.e., electrons to be disposed of) from the heart muscle under limiting oxygen conditions. In contrast, LDH-5, which is expressed in the liver, muscle and other tissues, has a higher K_M_ of about 0.29 mM for pyruvate. In addition, its K_M_ for NADH (0.17 mM) is higher than that of LDH-1 (0.04 mM). These higher K_M_ values of LDH-5 are supposed to support the disposal of electrons from the liver and other tissues only under “electron overflow” conditions, i.e., when the TCA cycle or biosynthetic pathways are sufficiently supplied with pyruvate, and oxidative phosphorylation provides enough ATP. Consistently, LDH-5 (LDH-A) was largely found to be up-regulated in many tumors [11,133,135,138], while LDH-1 (LDH-B) was found to be down-regulated in tumors [11,136]. However, for comparing the properties of LDH in tumor cells versus normal cells, only insufficient data are available. Only one study [124] showed that the K_M_ values of LDH-5, partially purified from normal and breast cancer patients, for pyruvate and NADH did not differ significantly, while the K_M_ values for lactate and NAD^+^ approximately doubled in tumor cells (see Table S5). The molecular basis for the latter effect was not specified in the study. However, it can be generally stated that the kinetic parameters of a given enzyme protein involved in pyruvate and lactate metabolism are essentially not changed by mutations to work differently in comparison to the very same enzyme in normal tissue. In contrast, the metabolic reprogramming in cancer cells is caused by gene expression changes of the “normal”, unchanged “wild-type” enzyme, by changing the relative abundances of its isoforms. In the case considered here, the normal wild-type LDH-5 (LDH-A) isoform is up-regulated in many tumors, and this wild-type LDH-5 isoform has a lower affinity (i.e., higher K_M_) for pyruvate compared to the normal wild-type LDH-1 (LDH-B) isoform, while these differences in the K_M_ for pyruvate are due to amino acid differences between isoforms that cause subtle changes in the active center [120].

In addition, the version of pyruvate kinase [71] that usually is expressed in fetal as well as in many normal adult tissues, PKM2, was reported to be up-regulated in cancer cells [9,144] and to be regulated in a positive feedback loop with HIF1 [145]. Even though it is widely assumed that PKM2 acts as a bottleneck in metabolite flux through glycolysis in cancer cells [146], this view is challenged by some reports, showing that PKM2 activity is not limiting for metabolite flux in cancer cells [57,147], and it is even dispensable for cancer cells [148]. For PKM2, K_M_ values for the enzymes from tumor cells were also reported (Table 2), but the results show that neither in the absence nor in the presence of F1,6BP do the K_M_ values for pyruvate essentially differ among the enzymes. This again suggests that changes in glycolysis rates in the investigated tumor cells are largely due to the expression changes mentioned above, but they are not due to the change in intrinsic enzyme kinetic parameters by mutation of the coding sequence of the enzyme. However, PKM2 is not the only pyruvate kinase found to be up-regulated in cancer cells. In some cases, the subtype of up-regulated PK was not specified [149], or PKL/R was found up-regulated [11,135], or PKM2 was explicitly shown not to be up-regulated [150].

Even the resulting increase in the actual metabolite concentrations in the cancerous cells compared to non-cancerous cells has been demonstrated by some studies, including metabolites of the TCA cycle, lactate, and amino acids [20,133]. Metabolite concentrations after Hepatitis C Virus (HCV)-associated reprogramming of hepatocellular metabolism were also analyzed, showing an increase in the concentrations of the metabolites of glycolysis [138], PPP [138], the TCA cycle [138], amino acids [138,151], fatty acid [138], and cholesterol biosynthesis [138].

## 5. Maintaining the Redox Balance under the Forced Growth Conditions in Cancer Cells

**Figure 3:**
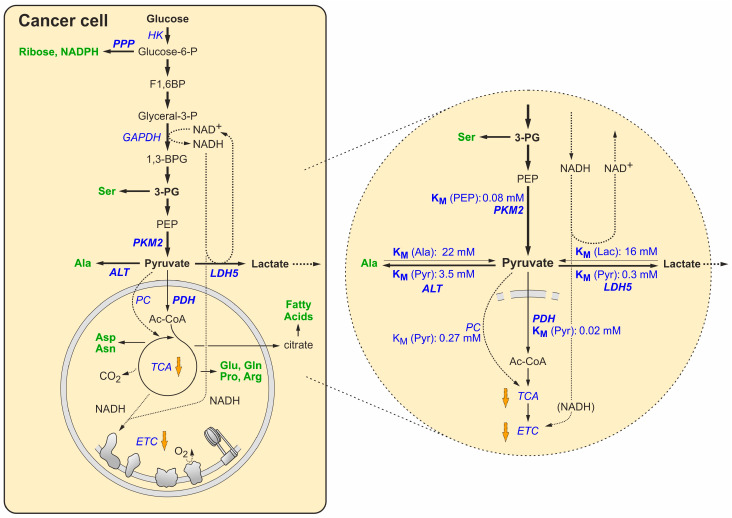
Changes in metabolite flux and maintenance of redox balance in growing cancer cells. This view is simplified to allow the reader to focus on metabolite flux at the pyruvate junction. The blow-up in the right part emphasizes the Michaelis constants (K_M_ values) of enzymes at the pyruvate junction (see Table 2), with the K_M_ value of PKM2 shown under stimulation by F1,6BP. Orange arrows pointing downwards indicate the slow-down of metabolite and electron flux through the TCA cycle and ETC/OXPHOS. PPP, pentose phosphate pathway; Ser, serine; Asp, aspartate; Asn, asparagine; Glu, glutamic acid; Gln, glutamine; Pro, proline; Arg, arginine.

Under conditions of sufficient supply of glucose both in normal and in cancer cells, carbon-body flux through glycolysis [26], the PDH reaction, and the TCA cycle, together with the downstream electron transport chain (and oxidative phosphorylation) in the mitochondrion, provide enough energy in form of ATP [27] that is required for cell growth. Thereby, the ATP yield from glycolysis in combination with PDH, TCA, and ETC/OXPHOS is about 16-fold higher than the ATP yield provided by the sole anaerobic glycolysis [27]. Accumulating mitochondrial ATP can then slow down the TCA cycle at the isocitrate dehydrogenase reaction, resulting in the accumulation of citrate, which can shuttle to the cytosol and provide acetyl-CoA for fatty acid synthesis [27,29,152,153]. Moreover, in any growing cells the synthesis of some non-essential amino acids and porphyrins also requires to withdraw metabolites from the TCA cycle [27,154] (see Figure 3). For the above reasons, it would absolutely not make sense for a growing cancer cell to completely shut down the TCA cycle and ETC/OXPHOS. This idea is strongly supported by the findings that mitochondrial function is largely retained among many tumors [20,21,155], and that suppression of mitochondrial function resulted in impaired tumorigenicity [22].

Consequently, in cancer cells, the TCA cycle and ETC/OXPHOS are only moderately slowed down by the gene regulatory mechanisms described above but not completely stopped, allowing the ongoing production of enough ATP and TCA metabolites required for growth. Concurrently, glycolysis and PPP flux are enhanced to allow increased withdrawal of metabolites that are required for growth as well, including amino acids, riboses, and NADPH (as detailed in Section 4).

Under normal conditions in non-cancerous cells, the electrons from cytosolic NADH are transported into the mitochondrion via the malate and glycerophosphate shuttles [27], entering the ETC/OXPHOS. Under these conditions, all electrons from the cytosolic NADH can be discharged to the ETC and are finally transferred to O_2_, with no need to discharge excess cytosolic electrons to pyruvate. In contrast, under the conditions of enhanced glycolytic flux in cancer cells, the cytosolic NAD^+^ is increasingly reduced to NADH in the GAPDH reaction, while the mitochondrial ETC/OXPHOS cannot accept all electrons from cytosolic NADH. Since the down-regulation of the TCA cycle and ETC/OXPHOS activities is largely managed by gene expression changes, the neat fine-tuning of these changes must be assumed to be slow, or even fail, compared to the need to correctly adjust the redox balance of the cell (i.e., getting rid of excess cytosolic electrons) in every single second in its life.

Only under these conditions of cytosolic electron accumulation, excess electrons from cytosolic NADH must be discharged and transferred to pyruvate, thereby forming lactate (Figure 3), which is then exported from the cancer cell in order to get rid of the excess electrons. In other words, lactate secretion from the tumor cell meets the short-term needs for electron disposal from cytosolic NADH to regenerate cytosolic NAD^+^, which is required to keep glycolysis running. From this view, nothing is enigmatic about lactate secretion from tumor cells; it just serves the short-term need to dispose of the excess cytosolic electrons.

These needs for providing both enough ATP and enough metabolites for cancer cell growth while maintaining the cytosolic redox balance (i.e., getting rid of excess electrons) are perfectly reflected by the K_M_ values of the enzymes at the pyruvate junction (please see the blow-up in Figure 3). Pyruvate dehydrogenase, the gate keeper for efficient downstream ATP synthesis via the TCA cycle and ETC/OXPHOS, has an extremely low K_M_ of 0.02 mM for pyruvate (Table 2), i.e., it binds pyruvate with very high affinity. By that, the K_M_ of PDH is much lower than intracellular pyruvate concentrations (Table 1). Just by these facts, we are informed about what is the most important pathway at the pyruvate junction in cancer cells. It is the efficient production of large amounts of ATP which are required for the increased growth demands of the cancer cell. Even if PDH kinases (PDKs) are overexpressed in many cancers [126], this essentially means that the PDH activity is adjusted more strictly to the metabolic requirements in the mitochondrion but not completely stopped.

The second important pathway deriving from the pyruvate junction is the LDH reaction. In contrast to red blood cells, which need to efficiently dispose of the electrons from NADH and therefore largely express LDH-1 (LDH-B) with a low K_M_ for pyruvate of 0.1 mM, cancer cells express LDH-5 (LDH-A) with a higher K_M_ of about 0.29 mM. This means that only accumulating pyruvate that is not used to feed the TCA cycle is available to accept excess “overflow” electrons and form lactate, which then disposes of the electrons from the cells by being secreted. Thus, we can call the LDH reaction the “emergency exit” for overflow electrons in the cytosol. Consistently, the K_M_ of LDH-5 for lactate, i.e., for the reverse reaction from lactate to pyruvate, is about 16 mM, i.e., about 50-fold higher than its K_M_ for pyruvate.

Ranked even behind LDH in the priority list for the use of pyruvate, ALT can convert pyruvate to alanine for biosynthesis purposes, with a K_M_ of 2.8 mM for pyruvate, i.e., again about 10-fold higher than the K_M_ of LDH. Thus, also in cancer cells, alanine is only synthesized from pyruvate when the needs for efficient ATP synthesis (by PDH, the TCA cycle and OXPHOS), as well as the maintenance of the cytosolic redox balance by LDH, are covered.

Taken together, the ranking list of pathways at the pyruvate junction in cancer cells is as follows: (1) the production of sufficient amounts of ATP via PDH, the TCA cycle, and ETC/OXPHOS; (2) maintenance of the cytosolic redox balance by disposing of overflow electrons via the LDH reaction; and (3) the use of pyruvate for the synthesis of oxaloacetate (PC reaction) and alanine (ALT reaction). Thereby, the secretion of lactate meets the short-term needs of the cell for maintaining the cytosolic redox balance, compensating possible imbalances between the up-regulation of glycolysis and the down-regulation of ETC/OXPHOS. Only beyond this, lactate secretion can become obligatory when O_2_ is actually in short supply, e.g., in tumors with insufficient vascularization.

The cotransport of lactate and protons by MCT-1—4 transporters takes place according to intracellular and extracellular lactate concentrations, since MCTs are energy-independent transporters [36]. However, the secretion of large amounts of lactate may lead to lactate acidosis. While this may be compensated in the body by changes in respiration, as well as by the balance between reduced hepatic urea formation and renal bicarbonate elimination versus increased renal NH_4_^+^ secretion, it is known for non-tumorous adipocytes that lactate inhibits glycolysis via signaling by the lactate receptor GPR81, resulting in a decrease in the cAMP concentration [156]. In contrast, in breast cancer cells, this negative feedback loop of lactate on glycolysis appears to be ineffective, since the knockdown of the lactate sensor GPR81 actually decreased expression of hexokinase 2, PFK-1, LDH-A, and MCT-4, as well as lactate secretion [157]. In turn, this means that the lactate sensing by the breast cancer cells causes an increased expression of lactate transporters and glycolysis enzymes (of these, LDH-A and MCT-4 are those isoenzymes with the highest K_M_ in their respective spectrum of isoenzymes), thereby supporting cancer cell growth irrespective of the acidification of the environment by lactate.

The above idea of using the LDH reaction as a temporary “emergency exit” for cytosolic overflow electrons only for a fraction, but not for all of the metabolized glucose, also makes sense when we consider the stoichiometry of carbon atoms during glucose breakdown. In glycolysis, one C_6_ glucose molecule is split into two C_3_ units (glyceraldehyde-3-phosphate), giving rise to two molecules of NADH [26]. If these two NADH molecules would serve to quantitatively produce two molecules of lactate from pyruvate in the LDH reaction, the cell would secrete stoichiometric amounts of lactate, but no carbon-body units would be left for any biosynthesis reactions deriving from the glycolysis metabolites downstream of glucose-6-phosphate. While these considerations do not apply to the PPP since it derives upstream of the GAPDH reaction, they apply in particular to lipid synthesis, which requires carbon input into the TCA cycle. Thus, quantitatively discharging the electrons from cytosolic NADH only to pyruvate (forming lactate) would be absolutely useless for a growing cancer cell.

Therefore, we must consider that a certain fraction of cytosolic NADH must always discharge its electrons in the mitochondrial ETC, since only then the remaining carbon-body backbones can be used for synthesizing lipids and certain amino acids. These various requirements must be continuously balanced and fine-tuned in the growing cell in a dynamic way to meet the requirements of both ATP production and biosyntheses. Thereby, the LDH enzymes help to keep the required redox balance in the cytosol in the cancer cells. Consequently, a complete LDH knockout [158,159] switches the cells to a “respiration only” phenotype (i.e., abolishes the Warburg effect) but does not kill the cells.

The notion that the LDH reaction is only used as an “emergency exit” for cytosolic overflow electrons does not mean that under the comfortable conditions of the supply of a tumor with plenty of glucose, lactate production cannot be wasteful. In the early experiments of Cori and Cori with chicken wing tumors (Table II in [4]), 23 mg of glucose resulted in the production of 16.2 mg of lactate. This means that on average, about 73% of the glucose entering a tumor was converted to lactate. Otto Warburg also found that a Jensen sarcoma converted about 68% of the incoming glucose into lactate (Tables II and IV in [5]). From a more recent tracer study, we can calculate that about 73% of glucose was converted into lactate by the cancer cells (calculated from the consumption of glucose and the production of lactate after 5 h, shown in the lower panel of Figure 2D in [68]). From an NMR study, we can calculate that about 52% of the input glucose had been converted to lactate in Huh-7 hepatoma cells (calculated from the data in Table 1 in [160]). In a study with about 80 non-small cell lung cancer cells lines, the authors found that both the extent of lactate secretion and the ratio of molecules of secreted lactate per molecules of input glucose largely varied among different cancer cell lines [161]. However, the above findings also make it sufficiently clear that not all of the incoming glucose is stoichiometrically converted to lactate (which, as detailed above, would not make sense for growing cells).

In turn, from the above considerations, we can also conclude that it is sufficient for the tumor cells to establish a relatively raw balance between the down-regulation of the disposal of electrons from cytosolic NADH through ETC/OXPHOS on the one hand, and the up-regulation of glucose uptake, glycolysis, and the withdrawal of carbon-body backbones for biosynthesis purposes on the other hand, since a quite large fraction of cytosolic overflow electrons can take the “emergency exit” to lactate.

## 6. Conclusions

Cancer cells reprogram their metabolism to meet the requirements of uncontrolled growth. The tumor cells derive from normal cells by several mutations that either knock down or knock out functions involved in growth control, as well as by mutations that often result in uncontrolled overactivities that promote cell proliferation. Thereby, the uptake and consumption of glucose by tumor cells is grossly increased to feed the PPP and the glycolysis with metabolites for biosynthesis reactions. At the same time, the downstream bottle-neck of the TCA cycle and OXPHOS is usually roughly kept in its normal activity state, or it is even down-regulated to allow the accumulation of upstream metabolites, which are then available for a massive increase in biosynthesis reactions for growth. Remarkably, the TCA cycle and OXPHOS activity are primarily maintained in cancer cells to allow efficient ATP production when oxygen is available. However, since such gross changes in gene expression and protein activities—at least those caused by somatic mutations—are usually not inherited, but often kill the host, we can assume that often there may have been no chance and also no need during evolution for a delicate fine-tuning of the balance between the above metabolic reprogramming events. Under the above conditions of up-regulated glycolysis, the production of NADH in the GAPDH reaction may therefore exceed the capacity of OXPHOS to re-oxidize cytosolic NADH to NAD^+^. Simply, this is the reason why cancer cells secrete lactate even under a sufficient oxygen supply (the phenomenon called the Warburg effect).

Careful inspection of the properties of the enzymes utilizing pyruvate in normal cells reveals that they are well-suited for that task in various tissues, as detailed in Section 2 and Section 3 above. In cancer cells, the properties of these enzymes at the pyruvate junction are not changed by cancer mutations but remain the same, while selected enzymes are expressed in isoenzyme variants with higher K_M_ values to increase metabolite concentrations in the cancer cells. One of them is LDH-5 (LDH-A), which is up-regulated in many cancer cells to ensure disposal of only overflow electrons by virtue of the higher K_M_ value of LDH-5 for pyruvate (0.29 mM), in comparison with that of its isoenzyme LDH-1 (0.1 mM). This keeps the pyruvate concentrations in the cancer cells high enough, both for efficient ATP production by the TCA cycle/OXPHOS and for biosynthesis reactions branching from the pyruvate junction. This is supported by changes in lactate transporter expression towards MCT-4 and MCT-1, which also have higher K_M_ values, to support the increase in intracellular metabolite concentrations.

In the combined view of both aspects—the uncontrolled cytosolic redox imbalance and the appropriate K_M_ values of the pyruvate junction enzymes—the above considerations provide a rather simple solution for a phenomenon that was regarded unsolved for decades of cancer research. Such a simple solution for a rather complex problem may appear counter-intuitive at first sight. However, the solution of a problem can be surprisingly more simple than it may be expected in relation to the importance of the problem [162]. For example, the complex task of regulating the blood glucose level in the body can be basically attributed to a simple property of a single enzyme, the K_M_ value of glucokinase expressed in the pancreatic β-cells and hepatocytes [26,163]. In a similar way, the K_M_ values of the enzymes at the pyruvate junction are appropriate to rescue the cytosolic redox balance in the cell, even under the rough conditions of rather unbalanced gene expression deregulation in cancer cells. Under such conditions, insufficient electron discharge in the respiratory chain can be compensated by disposing of cytosolic overflow electrons towards pyruvate, thereby taking the “emergency exit” by secreting lactate. However, pyruvate must also be available for other reactions, in particular pyruvate dehydrogenase and pyruvate carboxylase; therefore, the threshold for electron disposal using pyruvate should not be too low.

Accordingly, the first priority for the use of pyruvate in growing cancer cells is the efficient generation of ATP by the TCA cycle and OXPHOS—reflected by the very low K_M_ of PDH for pyruvate (K_M_ = 0.02 mM). The second priority is the maintenance of the redox balance in the cell by LDH (K_M_ = 0.1–0.29 mM). Equally importantly, pyruvate carboxylase (K_M_ = 0.265 mM) needs to refill the depleted TCA cycle metabolites. The production of alanine from pyruvate by ALT (K_M_ = 2.8 mM) is only the third priority. These enzymatic properties of the key enzymes at the pyruvate junction are in full accordance with lactate secretion from tumor cells being a consequent and required measure for maintaining the cytosolic redox balance by disposing the overflow electrons via lactate secretion.

According to the important role of lactate in tumor metabolism, the inhibition of LDHs and MCTs is considered an option in cancer therapy and is already used in preclinical and clinical trials [164,165]. Likely due to the important role of LDH-5 (LDH-A) in raising the intracellular pyruvate concentration in cancer cells by its higher K_M_ value (see above), LDH-5 expression correlates with a decrease in patient survival [166]. While the deletion of LDH-5 can partially inhibit carcinoma development [167], lactate secretion from cancer cells can be completely prevented only by the double knockout of LDH-A and LDH-B [158,159,166]. However, even the double knockout does not kill the cancer cells but just switches them to an aerobic phenotype with efficient ATP production via the TCA cycle and OXPHOS [158,159,166]. Therefore, the pharmacological inhibition of LDHs and MCTs may contribute to the limitation of cancer cell growth, but it appears unlikely that it will generally kill tumor cells.

In addition, the potential clinical use of LDH or MCT inhibitors may be considered with some caution not only because of known adverse effects [165], but also because of the role of lactate in the metabolism of many body tissues. As discussed above, lactate is produced as well as consumed by many tissues [61]. In the heart muscle, such inhibitors may cause critical states under conditions of fasting, oxygen limitation or exercise. In erythrocytes, such inhibitors may interfere with the obligatory disposal of electrons, and in the liver with the removal of erythrocytes’ lactate from the blood. Therefore, treatment with LDH or MCT inhibitors may be suspected to cause severe side effects.

## Figures and Tables

**Table 2:** K_M_ values of key enzymes at the pyruvate junction.

Enzyme	Subtype	Cell Type	Normal/ Tumor	Substrate	Allosteric Effector ^1^	K_M_ (mM) Means	K_M_ (mM) Range ^2^	K_M_ (mM)SD	*n* ^3^	References
PK	M1	muscle	normal	PEP	-	0.057	0.032–0.085	0.019	7	[76,77,78,79,80,81,82]
	M2	many	normal	PEP	-	0.421	0.140–0.860	0.223	8	[77,78,79,81,82,83,84,85]
			tumor	PEP	-	0.648	0.130–2.100	0.542	9	[57,86,87]
			normal	PEP	F1,6BP	0.064	0.030–0.100	0.026	4	[77,78,85,88]
			tumor	PEP	F1,6BP	0.103	0.030–0.170	0.057	3	[57,86,87]
	L, R	liver, RBC	normal	PEP	-	0.736	0.500–1.100	0.178	8	[75,78,79,81,88,89]
			normal	PEP	F1,6BP	0.073	0.060–0.090	0.013	3	[75,81]
PDH			normal	pyruvate	-	0.020	0.005–0.043	0.011	9	[90,91,92,93,94,95,96,97]
PC			normal	pyruvate	-	0.265	0.230–0.300	0.035	2	[98,99]
GPT/ ALT			normal	pyruvate	-	2.800	0.070–12.50	4.858	5	[100,101,102,103,104]
			normal	Ala	-	22.003	10.12–34.00	7.735	8	[100,101,102,103,104,105,106,107]
			normal	Glu	-	9.830	3.22–15.00	4.568	4	[101,102,103,107]
			normal	2-OG	-	0.523	0.100–1.100	0.356	6	[100,101,102,103,105,106]
LDH	1 (B, H)	heart, (RBC)	normal	pyruvate	-	0.100	0.034–0.243	0.063	17	[30,32,106,108,109,110,111,112,113,114,115,116,117,118,119,120]
			normal	lactate	-	5.916	2.000–9.690	2.911	8	[30,32,108,109,115,121,122]
			normal	NADH	-	0.038	0.014–0.069	0.023	3	[32,112,116]
			normal	NAD^+^	-	0.123	0.075–0.170	0.039	3	[30,109,121]
	5 (A, M)	liver, muscle, (heart) (RBC)	normal	pyruvate	-	0.288	0.095–0.630	0.173	12	[30,32,109,111,113,115,117,118,120,123,124]
			*normal ^4^*	*pyruvate*	*-*	*0.630*	*-*	*-*	*1 ^4^*	*[124]*
			*tumor ^4^*	*pyruvate*	*-*	*0.780*	*-*	*-*	*1 ^4^*	*[124]*
			normal	lactate	-	15.940	6.880–40.000	7.759	15	[30,32,109,115,121,122,124,125]
			*normal ^4^*	*lactate*	*-*	*10.730*	*-*	*-*	*1 ^4^*	*[124]*
			*tumor ^4^*	*lactate*	*-*	*21.780*	*-*	*-*	*1 ^4^*	*[124]*
			normal	NADH	-	0.173	0.016–0.330	0.157	2	[32,124]
			*normal ^4^*	*NADH*	*-*	*0.300*	*-*	*-*	*1 ^4^*	*[124]*
			*tumor ^4^*	*NADH*	*-*	*0.330*	*-*	*-*	*1 ^4^*	*[124]*
			normal	NAD^+^	-	0.337	0.220–0.500	0.119	3	[109,121,124]
			*normal*	*NAD^+^*	*-*	*0.500*	*0.220–0.500*	*0.119*	*3*	*[124]*
			*tumor*	*NAD^+^*		*0.990*	*-*	*-*	*1*	*[124]*

^1^ Stated only when applied for experimental K_M_ measurement. ^2^ As found over all the various references. Details are shown in Supplementary Tables S2–S5. ^3^ Number of independent experiments from references. Where available, data for tumor cells are shown separately from normal cells. ^4^ For one study [124], the value for normal cells is also displayed separately for direct comparison with its counterpart in tumor cells from the same study (in *italics*). Details are shown in Supplementary Tables S2 and S5. Abbreviations: GPT, glutamate pyruvate transaminase; GPT, glutamate oxoglutarate transaminase (=ALT); Ala, alanine; Glu, glutamate; 2-OG, 2-oxoglutarate (α-ketoglutarate).

## Supplementary Materials

The following supporting information can be downloaded at: https://www.mdpi.com/article/10.3390/cancers16132290/s1, Table S1: Experimentally determined substrate concentrations (related to Table 1); Table S2: Experimentally determined KM values of pyruvate kinase (PK) (related to Table 2); Table S3: Experimentally determined KM values of pyruvate dehydrogenase (PDH) and pyruvate carboxylase (PC) (related to Table 2); Table S4: Experimentally determined KM values of glutamate-pyruvate transaminase (GPT) (alanine transaminase, ALT) (related to Table 2); Table S5: Experimentally determined KM values of lactate dehydrogenase (LDH) (related to Table 2).
