# Supplementary material for: Importance of Michaelis Constants for Cancer Cell Redox Balance and Lactate Secretion—Revisiting the Warburg Effect
Source: Cancers (Basel). 2024 Jun 21;16(13):2290. doi: 10.3390/cancers16132290 (PMC11240417; doi:10.3390/cancers16132290)
Supplement: Supplementary file 1 [file cancers-16-02290-s001.zip › cancers-3071451-supplementary.pdf]

Supplementary information on: Importance of Michaelis constants for cancer cell redox balance and lactate secretion - revisiting the Warburg Effect, by Michael Niepmann

Table S1. Experimentally determined substrate concentrations (related to Table 1). Means, standard deviations ranges and number of measurements are given in Table 1 in the main text.

Sample	Metabolite	mM	cell (line)	cancer cell (line)?	References
**Blood**	**lactate** (resting)	**0.800**	-	-	[1]
		**1.190**	-	-	[2]
		**1.200**	-	-	[3]
		**1.200**	-	-	[4]
		**1.200**	-	-	[5]
		**1.200**	-	-	[6]
		**1.600**	-	-	[3]
		**2.000**	-	-	[7]
		**2.080**	-	-	[8]
		**3.500**	-	-	[9]
	**pyruvate** (resting)	**0.032**	-	-	[9]
		**0.045**	-	-	[10]
		**0.071**	-	-	[2]
		**0.075**	-	-	[11]
		**0.076**	-	-	[4]
		**0.090**	-	-	[8]
		**0.120**	-	-	[1]
	**lactate** (exercising)	**5.500**	-	-	[3]
		**5.500**	-	-	[4]
		**6.000**	-	-	[1]
		**6.500**	-	-	[11]
		**7.500**	-	-	[8]
**RBC (intracellular)**	**lactate**	**0.200**	RBC	-	[7]
		**0.940**	RBC	-	[2]
		**0.960**	RBC	-	[2]
		**0.964**	RBC	-	[12]
		**1.010**	RBC	-	[13]
		**1.200**	RBC	-	[5]
		**1.870**	RBC	-	[14]
	**pyruvate**	**0.043**	RBC	-	[2]
		**0.052**	RBC	-	[13]
		**0.063**	RBC	-	[14]
		**0.064**	RBC	-	[12]
		**0.083**	RBC	-	[2]
	**NAD^+**	**0.040**	RBC	-	[2]
		**0.062**	RBC	-	[13]
	**NADH**	**0.027**	RBC	-	[13]
**tumor cell lines (intracellular)**	**lactate**	**2.000**	4T1	yes	[15]
		**3.950**	SGC7901	yes	[15]
		**5.030**	RKO	yes	[15]
		**5.380**	Bcap37	yes	[15]
		**6.300**	HeLa	yes	[15]
		**6.860**	SMMC7721	yes	[15]
		**6.950**	HepG2	yes	[15]
		**15.000**	cervix cancer	yes	[16]
		**15.758**	NB-4	yes	[17]
		**20.400**	MC/CAR	yes	[17]
		**33.240**	HeLa S3	yes	[17]
		**35.525**	HEK293	yes	[17]
	**pyruvate**	**0.130**	A549	yes	[18]
		**0.148**	HeLa	yes	[19]
		**0.170**	MGC80-3	yes	[18]
		**0.180**	RKO	yes	[15]

		**0.200**	HepG2	yes	[15]
		**0.210**	HeLa	yes	[15]
		**0.220**	H1299	yes	[18]
		**0.229**	NB-4	yes	[17]
		**0.230**	Bcap37	yes	[15]
		**0.230**	RKO	yes	[18]
		**0.270**	SGC7901	yes	[15]
		**0.280**	4T1	yes	[15]
		**0.320**	SMMC7721	yes	[15]
		**0.330**	SK-HEP-1	yes	[18]
		**0.440**	HeLa	yes	[18]
		**0.707**	HEK293	yes	[17]
		**1.366**	HeLa S3	yes	[17]
		**2.390**	MC/CAR	yes	[17]
		**5.880**	iMBK (immortalized mouse kidney cells)	yes	[20]
	**NAD^+**	**0.470**	HeLa	yes	[19]
		**0.502**	iMBK	yes	[20]
	**NADH**	**0.065**	HeLa	yes	[19]
		**0.075**	iMBK	yes	[20]

Table S2. Experimentally determined K_M values of pyruvate kinase (PK) (related to Table 2). Means, standard deviations ranges and number of measurements are given in Table 2 in the main text.

Enzyme	Enzyme subtype, cell type	normal / tumor	Substrate	allosteric effector[1]	K_M (mM)	Reference
PK	**M1** (muscle)	normal	PEP	-	0.032	[21]
					0.033	[22]
					0.045	[23]
					0.065	[24]
					0.065	[25]
					0.075	[26]
					0.085	[27]
		tumor	PEP	-	-	-
	**M2** (many)	normal	PEP	-	0.140	[24]
					0.200	[27]
					0.300	[28]
					0.300	[25]
					0.450	[23]
					0.500	[26]
					0.620	[29]
					0.860	[30]
		tumor	PEP	-	0.130 (4T1)	[15]
					0.200	[31]
					0.460 (SMMC7721)	[15]
					0.520 (RKO)	[15]
					0.570 (Bcap37)	[15]
					0.590 (SGC7901)	[15]
					0.620 (HepG2)	[15]
					0.640 (HeLa)	[15]
					2.100	[32]
		normal	PEP	F1,6BP	0.030	[24]
					0.050	[26]
					0.075	[33]
					0.100	[30]
		tumor	PEP	F1,6BP	0.030	[15]
					0.110	[31]
					0.170	[32]
	**L, R** (liver, RBC)	normal	PEP	-	0.500	[34]
					0.550	[25]
					0.650	[25]
					0.660	[35]
					0.790	[27]
					0.800	[26]
					0.840	[33]
					1.100	[34]
		tumor	PEP	-	-	-
		normal	PEP	F1,6BP	0.060	[25]
					0.069	[35]
					0.090	[25]
		tumor	PEP	F1,6BP	-	-

[1] stated only when applied for experimental K_M measurement. Values are sorted by increasing metabolite concentrations values rather than by publication date. For tumor cells, data were available only for PKM2.

Table S3. Experimentally determined K_M values of pyruvate dehydrogenase (PDH) and pyruvate carboxylase (PC) (related to Table 2). Means, standard deviations ranges and number of measurements are given in Table 2 in the main text.

Enzyme	normal / tumor	Substrate	K_M (mM)	Reference
PDH	normal	pyruvate	0.005	[36]
			0.007	[37]
			0.010	[38]
			0.017	[39]
			0.022	[40]
			0.025	[41]
			0.025	[42]
			0.027	[39]
			0.043	[43]
	tumor	pyruvate	-	-
PC	normal	pyruvate	0.230	[44]
			0.300	[45]
	tumor	pyruvate	-	-

Values are sorted by increasing K_M values rather than by publication date. For tumor cells, no data were available.

Table S4. Experimentally determined K_M values of glutamate-pyruvate transaminase (GPT) (alanine transaminase, ALT) (related to Table 2). Means, standard deviations ranges and number of measurements are given in Table 2 in the main text.

Enzyme	normal / tumor	Substrate	K_M (mM)	Reference
GPT/ ALT	normal	pyruvate	0.070	[46]
			0.230	[47]
			0.300	[48]
			0.900	[49]
			12.500	[50]
	tumor	pyruvate	-	-
	normal	Ala	10.120	[51]
			13.000	[47]
			16.000	[46]
			21.900	[52]
			26.500	[52]
			26.500	[50]
			28.000	[48, 53]
			34.000	[49]
	tumor	Ala	-	-
	normal	Glu	3.220	[51]
			8.100	[47]
			13.000	[50]
			15.000	[49]
	tumor	Glu	-	-
	normal	2-OG	0.100	[50]
			0.120	[47]
			0.400	[48, 53]
			0.690	[52]
			0.730	[52]
			1.100	[49]
	tumor	2-OG	-	-

Values are sorted by increasing K_M values rather than by publication date. For tumor cells, no data were available.

Table S5. Experimentally determined K_M values of lactate dehydrogenase (LDH) (related to Table 2). Means, standard deviations ranges and number of measurements are given in Table 2 in the main text.

Enzyme	subtype	normal / tumor	Substrate	K_M (mM)	Reference
LDH	1 (B, H) heart, (RBC)	normal	pyruvate	0.034	[54]
				0.035	[55]
				0.038	[56]
				0.047	[57]
				0.050	[58]
				0.050	[59]
				0.050	[60]
				0.066	[61]
				0.080	[62]
				0.080	[63]
				0.120	[64]
				0.136	[65]
				0.140	[62]
				0.160	[52]
				0.172	[66]
				0.200	[67]
				0.243	[68]
		tumor	pyruvate	-	-
		normal	lactate	2.000	[54]
				2.600	[57]
				3.960	[69]
				4.100	[64]
				7.000	[62]
				8.980	[66]
				9.000	[62]
				9.690	[70]
		tumor	lactate	-	-
		normal	NADH	0.014	[57]
				0.030	[67]
				0.069	[65]
		tumor	NADH	-	-
		normal	NAD^+	0.075	[62]
				0.130	[64]
				0.170	[69]
		tumor	NAD^+	-	-

Values are sorted by increasing K_M values rather than by publication date. For tumor cells, no data were available.

Table S5 (continued). Experimentally determined K_M values of lactate dehydrogenase (LDH) (related to Table 2).

Enzyme	subtype	normal / tumor	Substrate	K_M (mM)	Reference
**LDH**	**5 (A, M)** liver, muscle, (heart) (RBC)	**normal**	pyruvate	0.095	[55]
				0.110	[58]
				0.110	[60]
				0.130	[57]
				0.150	[63]
				0.260	[66]
				0.275	[61]
				0.320	[62]
				0.400	[71]
				0.460	[64]
				0.520	[62]
				0.630[1]	***[72]***
		**tumor**	pyruvate	***0.780***[1]	***[72]***
		**normal**	lactate	6.880	[69]
				8.620	[73]
				10.500	[70]
				10.730[1]	***[72]***
				11.900	[73]
				13.500	[73]
				14.200	[57]
				14.300	[64]
				14.800	[70]
				14.800	[73]
				16.300	[73]
				17.600	[73]
				19.970	[66]
				25.000	[62]
				40.000	[62]
		**tumor**	lactate	***21.780***[1]	***[72]***
		**normal**	NADH	0.016	[57]
				0.300[1]	***[72]***
		**tumor**	NADH	***0.330***[1]	***[72]***
		**normal**	NAD^+	0.220	[64]
				0.290	[69]
				0.500[1]	***[72]***
		**tumor**	NAD^+	***0.990***[1]	***[72]***

Values are sorted by increasing K_M values rather than by publication date. [1] For tumor cells, only one study provided values for normal vs. tumor cells (Talaiezadeh et al., 2015), these values are in ***bold italics*** and are best compared only to the corresponding value from the same paper.

References

1. Carter, H.; Jones, A. M.; Doust, J. H., Changes in blood lactate and pyruvate concentrations and the lactate-to-pyruvate ratio during the lactate minimum speed test. *J Sports Sci* **2000,** 18, (3), 213-25.
2. Minakami, S.; Suzuki, C.; Saito, T.; Yoshikawa, H., Studies on erythrocyte glycolysis. I. Determination of the glycolytic intermediates in human erythrocytes. *J Biochem* **1965,** 58, (6), 543-50.
3. Hurley, B. F.; Hagberg, J. M.; Allen, W. K.; Seals, D. R.; Young, J. C.; Cuddihee, R. W.; Holloszy, J. O., Effect of training on blood lactate levels during submaximal exercise. *J Appl Physiol Respir Environ Exerc Physiol* **1984,** 56, (5), 1260-4.
4. Pianosi, P.; Seargeant, L.; Haworth, J. C., Blood lactate and pyruvate concentrations, and their ratio during exercise in healthy children: developmental perspective. *Eur J Appl Physiol Occup Physiol* **1995,** 71, (6), 518-22.
5. Hildebrand, A.; Lormes, W.; Emmert, J.; Liu, Y.; Lehmann, M.; Steinacker, J. M., Lactate concentration in plasma and red blood cells during incremental exercise. *Int J Sports Med* **2000,** 21, (7), 463-8.
6. Goodwin, M. L.; Harris, J. E.; Hernandez, A.; Gladden, L. B., Blood lactate measurements and analysis during exercise: a guide for clinicians. *J Diabetes Sci Technol* **2007,** 1, (4), 558-69.
7. Kohler, G.; Rost, F.; Seelig, J., Simultaneous separation of intracellular and extracellular lactate NMR signals of human erythrocytes. *Magn Reson Med* **2007,** 58, (2), 213-7.
8. Shinde, S.; Golam, K.; Kumar, P.; Patil, N.; Sadacharan, K., Perioperative blood lactate levels, pyruvate levels, and lactate-pyruvate ratio in children undergoing cardiopulmonary bypass for congenital heart disease. *Indian J. Crit. Care Med* **2005,** 9, 145-150.
9. Ceperuelo-Mallafre, V.; Reverte, L.; Peraire, J.; Madeira, A.; Maymo-Masip, E.; Lopez-Dupla, M.; Gutierrez-Valencia, A.; Ruiz-Mateos, E.; Buzon, M. J.; Jorba, R.; Vendrell, J.; Auguet, T.; Olona, M.; Vidal, F.; Rull, A.; Fernandez-Veledo, S., Circulating pyruvate is a potent prognostic marker for critical COVID-19 outcomes. *Front Immunol* **2022,** 13, 912579.
10. Landon, J.; Fawcett, J. K.; Wynn, V., Blood pyruvate concentration measured by a specific method in control subjects. *J Clin Pathol* **1962,** 15, (6), 579-84.
11. Freund, H.; Marbach, J.; Ott, C.; Lonsdorfer, J.; Heitz, A.; Zouloumian, P.; Kehayoff, P., Blood pyruvate recovery curves after short heavy submaximal exercise in man. *Eur J Appl Physiol Occup Physiol* **1980,** 43, (1), 83-91.
12. Niessner, H.; Beutler, E., Fluorometric analysts of glycolytic intermediates in human red blood cells. *Biochem Med* **1973,** 8, (1), 123-34.
13. Marshall, W. E.; Omachi, A., Measured and calculated NAD+-NADH ratios in human erythrocytes. *Biochim Biophys Acta* **1974,** 354, (1), 1-10.
14. Cavaliere, F.; Meo, F.; Scapigliati, A.; Sciarra, M.; Schiavello, R., Red blood cell energy metabolism during cardiopulmonary bypass. *J Cardiovasc Surg (Torino)* **1999,** 40, (5), 653-7.
15. Xie, J.; Dai, C.; Hu, X., Evidence That Does Not Support Pyruvate Kinase M2 (PKM2)-catalyzed Reaction as a Rate-limiting Step in Cancer Cell Glycolysis. *J. Biol. Chem.* **2016,** 291, (17), 8987-99.
16. Vaupel, P.; Multhoff, G., Revisiting the Warburg effect: historical dogma versus current understanding. *J Physiol* **2021,** 599, (6), 1745-1757.
17. Rost, L. M.; Brekke Thorfinnsdottir, L.; Kumar, K.; Fuchino, K.; Eide Langorgen, I.; Bartosova, Z.; Kristiansen, K. A.; Bruheim, P., Absolute Quantification of the Central Carbon Metabolome in Eight Commonly Applied Prokaryotic and Eukaryotic Model Systems. *Metabolites* **2020,** 10, (2).
18. Jin, C.; Hu, W.; Wang, Y.; Wu, H.; Zeng, S.; Ying, M.; Hu, X., The thermodynamic state of aerobic glycolytic flux plays a critical role in stabilizing aerobic glycolytic flux of cancer cells. *bioRxiv* **2022**.
19. Chen, W. W.; Freinkman, E.; Wang, T.; Birsoy, K.; Sabatini, D. M., Absolute Quantification of Matrix Metabolites Reveals the Dynamics of Mitochondrial Metabolism. *Cell* **2016,** 166, (5), 1324-1337 e11.
20. Park, J. O.; Rubin, S. A.; Xu, Y. F.; Amador-Noguez, D.; Fan, J.; Shlomi, T.; Rabinowitz, J. D., Metabolite concentrations, fluxes and free energies imply efficient enzyme usage. *Nat Chem Biol* **2016,** 12, (7), 482-9.
21. Reynard, A. M.; Hass, L. F.; Jacobsen, D. D.; Boyer, P. D., The correlation of reaction kinetics and substrate binding with the mechanism of pyruvate kinase. *J Biol Chem* **1961,** 236, 2277-83.
22. Baranowska, B.; Baranowski, T., Kinetic properties of human muscle pyruvate kinase. *Mol Cell Biochem* **1982,** 45, (2), 117-25.
23. Consler, T. G.; Woodard, S. H.; Lee, J. C., Effects of primary sequence differences on the global structure and function of an enzyme: a study of pyruvate kinase isozymes. *Biochemistry* **1989,** 28, (22), 8756-64.
24. van Berkel, T. J.; Koster, J. F.; Hulsmann, W. C., Some kinetic properties of the allosteric M-type pyruvate kinase from rat liver; influence of pH and the nature of amino acid inhibition. *Biochim Biophys Acta* **1973,** 321, (1), 171-80.
25. Hall, E. R.; Cottam, G. L., Isozymes of pyruvate kinase in vertebrates: their physical, chemical, kinetic and immunological properties. *Int J Biochem* **1978,** 9, (11), 785-93.
26. van Berkel, T. J. C. Pyruvate Kinase Isoenzymes. Proefschrift, Erasmus Universiteit te Rotterdam, 1974.
27. Eigenbrodt, E.; Schoner, W., Purification and properties of the pyruvate kinase isoenzymes type L and M2 from chicken liver. *Hoppe Seylers Z Physiol Chem* **1977,** 358, (8), 1033-46.

28. van Berkel, J. C., Some kinetic properties of M2-type pyruvate kinase from rat liver at physiological Mg2+ concentration. *Biochim Biophys Acta* **1974,** 370, (1), 140-52.
29. van Berkel, T. J.; de Jonge, H. R.; Koster, J. F.; Hulsmann, W. C., Kinetic evidence for the presence of two forms of M2-type pyruvate kinase in rat small intestine. *Biochem Biophys Res Commun* **1974,** 60, (1), 398-405.
30. Morgan, H. P.; O'Reilly, F. J.; Wear, M. A.; O'Neill, J. R.; Fothergill-Gilmore, L. A.; Hupp, T.; Walkinshaw, M. D., M2 pyruvate kinase provides a mechanism for nutrient sensing and regulation of cell proliferation. *Proc Natl Acad Sci U S A* **2013,** 110, (15), 5881-6.
31. Liu, V. M.; Howell, A. J.; Hosios, A. M.; Li, Z.; Israelsen, W. J.; Vander Heiden, M. G., Cancer-associated mutations in human pyruvate kinase M2 impair enzyme activity. *FEBS Lett* **2020,** 594, (4), 646-664.
32. Dombrauckas, J. D.; Santarsiero, B. D.; Mesecar, A. D., Structural basis for tumor pyruvate kinase M2 allosteric regulation and catalysis. *Biochemistry* **2005,** 44, (27), 9417-29.
33. Tanaka, T.; Harano, Y.; Sue, F.; Morimura, H., Crystallization, characterization and metabolic regulation of two types of pyruvate kinase isolated from rat tissues. *J Biochem* **1967,** 62, (1), 71-91.
34. Koster, J. F.; Slee, R. G.; Staal, G. E.; van Berkel, T. J., The influence of glucose I,6-diphosphate on the enzymatic activity of pyruvate kinase. *Biochim Biophys Acta* **1972,** 258, (3), 763-8.
35. Hubbard, D. R.; Cardenas, J. M., Kinetic properties of pyruvate kinase hybrids formed with native type L and inactivated type M subunits. *J Biol Chem* **1975,** 250, (13), 4931-6.
36. Fang, R.; Nixon, P. F.; Duggleby, R. G., Identification of the catalytic glutamate in the E1 component of human pyruvate dehydrogenase. *FEBS Lett* **1998,** 437, (3), 273-7.
37. Wu, Y. G.; Chen, W. Y.; Zhang, Z. W.; Yang, G. Z.; Li, W.; Duggleby, R. G., Biochemical characterization of two mutants of human pyruvate dehydrogenase, F205L and T231A of the E1alpha subunit. *J Inherit Metab Dis* **2003,** 26, (7), 671-4.
38. Seifert, F.; Golbik, R.; Brauer, J.; Lilie, H.; Schroder-Tittmann, K.; Hinze, E.; Korotchkina, L. G.; Patel, M. S.; Tittmann, K., Direct kinetic evidence for half-of-the-sites reactivity in the E1 component of the human pyruvate dehydrogenase multienzyme complex through alternating sites cofactor activation. *Biochemistry* **2006,** 45, (42), 12775-85.
39. Blass, J. P.; Lewis, C. A., Kinetic properties of the partially purified pyruvate dehydrogenase complex of ox brain. *Biochem J* **1973,** 131, (1), 31-7.
40. Czygier, M.; Strumilo, S. A., Basic properties of the pyruvate dehydrogenase complex isolated from aurochs heart. *Acta Biochim Pol* **1994,** 41, (4), 453-7.
41. Kiselevsky, Y. V.; Ostrovtsova, S. A.; Strumilo, S. A., Kinetic characterization of the pyruvate and oxoglutarate dehydrogenase complexes from human heart. *Acta Biochim Pol* **1990,** 37, (1), 135-9.
42. Liu, S.; Gong, X.; Yan, X.; Peng, T.; Baker, J. C.; Li, L.; Robben, P. M.; Ravindran, S.; Andersson, L. A.; Cole, A. B.; Roche, T. E., Reaction mechanism for mammalian pyruvate dehydrogenase using natural lipoyl domain substrates. *Arch Biochem Biophys* **2001,** 386, (2), 123-35.
43. Pavlu-Pereira, H.; Lousa, D.; Tome, C. S.; Florindo, C.; Silva, M. J.; de Almeida, I. T.; Leandro, P.; Rivera, I.; Vicente, J. B., Structural and functional impact of clinically relevant E1alpha variants causing pyruvate dehydrogenase complex deficiency. *Biochimie* **2021,** 183, 78-88.
44. Jitrapakdee, S.; Walker, M. E.; Wallace, J. C., Functional expression, purification, and characterization of recombinant human pyruvate carboxylase. *Biochem Biophys Res Commun* **1999,** 266, (2), 512-7.
45. Barash, V.; Khassis, S.; Granat, M.; Gutman, A., Pyruvate carboxylase activity in chorionic villi: possibility of application to prenatal diagnosis. *Enzyme* **1988,** 39, (1), 54-9.
46. Peracchi, A.; Polverini, E., Using Steady-State Kinetics to Quantitate Substrate Selectivity and Specificity: A Case Study with Two Human Transaminases. *Molecules* **2022,** 27, (4).
47. Bulos, B.; Handler, P., Kinetics of Beef Heart Glutamic-Alanine Transaminase. *J Biol Chem* **1965,** 240, 3283-94.
48. Saier, M. H., Jr.; Jenkins, W. T., Alanine aminotransferase. I. Purification and properties. *J. Biol. Chem.* **1967,** 242, (1), 91-100.
49. Hopper, S.; Segal, H. L., Kinetic studies of rat liver glutamicalanine transaminase. *J Biol Chem* **1962,** 237, 3189-95.
50. McAllister, C. H.; Facette, M.; Holt, A.; Good, A. G., Analysis of the enzymatic properties of a broad family of alanine aminotransferases. *PLoS One* **2013,** 8, (2), e55032.
51. Mu, X.; Qi, L.; Qiao, J.; Zhang, H.; Ma, H., Study on alanine aminotransferase kinetics by microchip electrophoresis. *Anal Biochem* **2012,** 421, (2), 499-505.
52. Bergmeyer, H. U.; Scheibe, P.; Wahlefeld, A. W., Optimization of methods for aspartate aminotransferase and alanine aminotransferase. *Clin. Chem.* **1978,** 24, (1), 58-73.
53. Saier, M. H., Jr.; Jenkins, W. T., Alanine aminotransferase. II. The basis for substrate specificity. *J Biol Chem* **1967,** 242, (1), 101-8.
54. Hakala, M. T.; Glaid, A. J.; Schwert, G. W., Lactic dehydrogenase. II. Variation of kinetic and equilibrium constants with temperature. *J Biol Chem* **1956,** 221, (1), 191-209.
55. Pasti, A. P.; Rossi, V.; Di Stefano, G.; Brigotti, M.; Hochkoeppler, A., Human lactate dehydrogenase A undergoes allosteric transitions under pH conditions inducing the dissociation of the tetrameric enzyme. *Biosci Rep* **2022,** 42, (1).

56. Boland, M. J.; Gutfreund, H., Pig heart lactate dehydrogenase. Binding of pyruvate and the interconversion of pyruvate-containing ternary complexes. *Biochem J* **1975,** 151, (3), 715-27.
57. Goto, T.; Sugawara, K.; Nakamura, S.; Kidokoro, S. I.; Wakui, H.; Nunomura, W., Enzymatic and thermodynamic profiles of a heterotetramer lactate dehydrogenase isozyme in swine. *Biochem Biophys Res Commun* **2016,** 479, (4), 860-867.
58. Nitisewojo, P.; Hultin, H. O., A comparison of some kinetic properties of soluble and bound lactate dehydrogenase isoenzymes at different temperatures. *Eur J Biochem* **1976,** 67, (1), 87-94.
59. Nadeem, M. S.; Moran, J.; Murtaza, B. N.; Muhammad, K.; Ahmad, H., Cloning, E. coli expression, and characterization of heart lactate dehydrogenase B from river buffalo (Bubalus bubalis). *Anim Biotechnol* **2014,** 25, (1), 23-34.
60. Read, J. A.; Winter, V. J.; Eszes, C. M.; Sessions, R. B.; Brady, R. L., Structural basis for altered activity of M- and H-isozyme forms of human lactate dehydrogenase. *Proteins* **2001,** 43, (2), 175-85.
61. Fernandez-Velasco, D. A.; Garza-Ramos, G.; Ramirez, L.; Shoshani, L.; Darszon, A.; Tuena de Gomez-Puyou, M.; Gomez-Puyou, A., Activity of heart and muscle lactate dehydrogenases in all-aqueous systems and in organic solvents with low amounts of water. Effect of guanidine chloride. *Eur. J. Biochem.* **1992,** 205, (2), 501-8.
62. Everse, J.; Kaplan, N. O., Lactate dehydrogenases: structure and function. *Adv Enzymol Relat Areas Mol Biol* **1973,** 37, 61-133.
63. Rao, Y.; Gammon, S. T.; Sutton, M. N.; Zacharias, N. M.; Bhattacharya, P.; Piwnica-Worms, D., Excess exogenous pyruvate inhibits lactate dehydrogenase activity in live cells in an MCT1-dependent manner. *J Biol Chem* **2021,** 297, (1), 100775.
64. Nisselbaum, J. S.; Packer, D. E.; Bodansky, O., Comparison of the Actions of Human Brain, Liver, and Heart Lactic Dehydrogenase Variants on Nucleotide Analogues and on Substrate Analogues in the Absence and in the Presence of Oxalate and Oxamate. *J Biol Chem* **1964,** 239, 2830-4.
65. Mali, A. V.; Bhise, S. S.; Katyare, S. S.; Hegde, M. V., Altered Kinetics Properties of Erythrocyte Lactate Dehydrogenase in Type II Diabetic Patients and Its Implications for Lactic Acidosis. *Indian J Clin Biochem* **2018,** 33, (1), 38-45.
66. Wang, Y.; Wei, L.; Wei, D.; Li, X.; Xu, L.; Wei, L., Enzymatic Kinetic Properties of the Lactate Dehydrogenase Isoenzyme C(4) of the Plateau Pika (Ochotona curzoniae). *Int J Mol Sci* **2016,** 17, (1).
67. Aragon, J. J.; Feliu, J. E.; Frenkel, R. A.; Sols, A., Permeabilization of animal cells for kinetic studies of intracellular enzymes: in situ behavior of the glycolytic enzymes of erythrocytes. *Proc Natl Acad Sci U S A* **1980,** 77, (11), 6324-8.
68. Al-Kasimy, L. N.; Al Jebur, L. A.; Maher, F. T., A Kinetic and Thermodynamic Study of The Enzyme Lactate Dehydrogenase Purified from Cardiac Patients' Blood. *HIV Nursing* **2023,** 23, (3), 1257–1266-1257–1266.
69. Buhl, S. N.; Jackson, K. Y.; Vanderlinde, R. E., The effect of temperature on the kinetic constants of human lactate dehydrogenase 1 and 5. *Clin Chim Acta* **1977,** 80, (2), 265-70.
70. Nakae, Y.; Stoward, P. J., The diverse Michaelis constants and maximum velocities of lactate dehydrogenase in situ in various types of cell. *Histochem J* **1994,** 26, (4), 292-7.
71. Pettit, S. M.; Nealon, D. A.; Henderson, A. R., Purification of lactate dehydrogenase isoenzyme-5 from human liver. *Clin Chem* **1981,** 27, (1), 88-93.
72. Talaiezadeh, A.; Shahriari, A.; Tabandeh, M. R.; Fathizadeh, P.; Mansouri, S., Kinetic characterization of lactate dehydrogenase in normal and malignant human breast tissues. *Cancer Cell Int* **2015,** 15, 19.
73. Nakae, Y.; Stoward, P. J., Kinetic parameters of lactate dehydrogenase in liver and gastrocnemius determined by three quantitative histochemical methods. *J Histochem Cytochem* **1997,** 45, (10), 1427-31.
